# Somatic mutant clone screening: scan for novel NASH target genes in mouse liver

**DOI:** 10.1038/s41392-023-01572-8

**Published:** 2023-08-25

**Authors:** Tian Lan, Adrien Guillot, Frank Tacke

**Affiliations:** 1 Lab of Gastroenterology and Hepatology, State Key Laboratory of Biotherapy, West China Hospital, Sichuan University, Chengdu, China; 2 Department of Gastroenterology, West China Hospital, Sichuan University, Chengdu, China; 3 Department of Hepatology & Gastroenterology, Charité - Universitätsmedizin Berlin, Campus Virchow-Klinikum and Campus Charité Mitte, Berlin, Germany

**Keywords:** Metabolic disorders, Genetic techniques

**Fig. 1:**
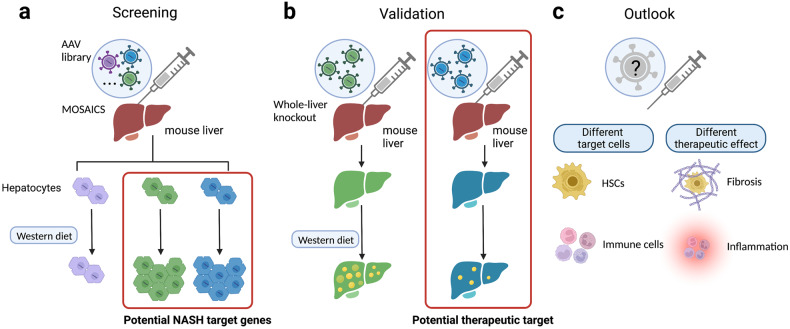
A novel method of in vivo hepatocyte clonal screening for non-alcoholic steatohepatitis (NASH) target genes in mouse liver. **a** In a recent *Cell* paper by Wang and colleagues, an Adeno-associated virus (AAV) library was created and injected into mouse liver to generate mosaic liver with multiple mutant hepatocyte clones followed by western diet-induced NASH. Target genes related to NASH pathogenesis were screened by measuring clonal expansion. **b** Whole-liver knockout mice were generated to measure the effect of the deletion of NASH target genes at the tissue or organ level. **c** Screening for inflammation/fibrosis target genes in hepatic stellate cells (HSCs) or immune cells is a potential future direction to discover more comprehensive therapeutic options for liver diseases. MOSAICS, method of somatic adeno-associated virus-transposon in vivo clonal screening. This figure was created with BioRender.com

In a landmark study recently published in *Cell*, Wang et al. demonstrated a novel method of somatic adeno-associated virus (AAV)-transposon in vivo clonal screening (termed MOSAICS), which is capable to generate multiple mutant hepatocyte clones within the same tissue and thereby to scan for non-alcoholic steatohepatitis (NASH) target genes (Fig. 1).^1^

Non-alcoholic fatty liver disease (NAFLD) is the most common liver disease affecting about 20–30% of the global population and associated with metabolic dysfunction. NAFLD can progress to NASH, characterized by liver inflammation, steatosis and hepatocyte ballooning, and NAFLD/NASH can lead to fibrosis, cirrhosis, and ultimately liver cancer. For decades, researchers have been seeking to understand its pathogenesis and to discover therapeutic strategies to treat NAFLD/NASH. However, up to now, there is still no specific pharmacotherapy available.

Somatic mutation is a non-heritable gene mutation that occurs in aged or chronically injured somatic cells, and this phenomenon is also observed in NAFLD patient livers. It has been suggested that somatic mutations are an adaptive mechanism that renders the mutated cells (partially) resistant to insults.^2^ Therefore, MOSAICS as an experimental approach to screen for somatic mutations that protect cells from metabolic injury in NAFLD could be valuable to uncover potential therapeutic targets. The unique vector designed for MOSAICS made it possible to quantify the single guide RNA (sgRNA) expression that is integrated into the cell genome and thereby measuring clone expansion. Utilizing this method, the authors found several previously undiscovered NASH-related genes, the deletion of which prevented hepatocytes from western diet-induced cell injury and improved liver steatosis.^1^

As a start, the authors tested the effect of somatic mutation of a well-known NASH-related gene, membrane bound o-acyltransferase 7 (*Mboat7*), in western diet-induced mouse NASH models. They first identified that whole-liver knockout of *Mboat7* exacerbated steatosis and liver injury.^1^ Next, to mimic somatic mutations in the liver, they generated small clones of *Mboat7*^*−/−*^ hepatocytes marked by fluorescent proteins by low-dose injection of AAV serotype 8-TBG-Cre. They found that these mutant clones were more vulnerable to the western diet-induced injury, and the clone size decreased dramatically, indicating that cells with these somatic mutations do show different responsiveness to injury.^1^

To discover somatic mutations in fatty liver in a more high-throughput way, the authors screened out 63 candidate genes from the sequencing database that might play a role in the pathogenesis of NASH. Then, they designed sgRNA libraries and generated somatic mosaic livers with mutant clones of all the candidate genes in each mouse followed by NASH modeling. By measuring clone expansion and sgRNA expression, the authors identified five top-ranked genes (*Acvr2a*, *Gpam*, *Dgat2*, *Srebf1*, and *Irs1*) that promoted clone expansion when deleted.^1^ These genes have been found involved in lipid metabolism and pathogenesis of fatty liver diseases.

In order to unearth more unanticipated genes involved in NASH, the authors broadly screened for transcription and epigenetic factors and discovered 472 factors whose expression and activities were changed in NAFLD/NASH and hepatitis C virus patient cohorts. Accordingly, the authors created sgRNA libraries and generated mosaic liver. After NASH modeling, 23 genes were revealed as potential contributors to the pathogenesis of NAFLD/NASH.^1^ Surprisingly, most of these genes have not been linked with fatty liver diseases before.

Although somatic mutations impacted the susceptibility of mutant hepatocyte clones to lipotoxicity in NASH models, it is unknown whether these cellular or clonal mutations have the same effect at the tissue or organ level. To detect whether the whole-liver deletion of these non-canonical NASH genes can achieve amelioration of liver steatosis, the authors generated 20 knockout mouse strains with the deletion of each of the transcription or epigenetic regulators of interest. Among them, over half presented with decreased liver weight upon challenge with western diet. Surprisingly, knockout of *Tbx3* (transcription factors) and *Smyd2* (epigenetic factor) resulted in the most significant reduction of liver injury and steatosis, even though *Tbx3* and *Smyd2* have not been linked to NAFLD/NASH pathogenesis before. RNA-sequencing further disclosed altered hepatic gene expression profiles, which partly overlapped in the liver of these knockout mice, especially in lipid metabolism, inflammation and fibrosis pathways.^1^ Finally, to verify the therapeutic potential of these newly discovered NASH genes, the authors tested the effect of AZ505, a selective SMYD2 inhibitor, in the western diet mouse model. Intraperitoneal administration of AZ505 significantly reduced liver mass and improved liver steatosis compared to the control group.^1^

Taken together, the authors described an innovative in vivo platform, MOSAICS, to generate mosaic liver harboring various somatic mutant hepatocyte clones. They applied this platform to screen genes that affect clonal fitness in NASH models and validated the effect on the NASH phenotype in whole-liver knockout mice for selected gene targets. These findings may indeed facilitate the identification of new therapeutic targets and strategies. However, by design, this approach will primarily identify genetic and epigenetic pathways related to metabolic injury in hepatocytes, while important pathogenic pathways activated in non-parenchymal cells—e.g., the matrix-producing activated hepatic stellate cells as main drivers of liver fibrosis—and/or in immune cells, which define the transition from steatosis to NASH, will remain concealed.^3^ In fact, deleting the discovered genes significantly reduced liver steatosis, but showed rather limited effects on liver fibrosis.^1^ Antifibrotic therapy for NASH is challenging yet crucial, as inflammation and fibrosis are main drivers of patient-related outcomes in NAFLD/NASH.^4^ Thus, targeting a distinct pathway globally in the liver may not be suitable to substantially halt inflammation or fibrosis, as compared to cell-type specific approaches. Similarly, deleting a gene (or gene variant) in the liver may have systemic consequences, as demonstrated for the natural E167K variant of *TM6SF2*. This *TM6SF2* variant favors lipid accumulation in hepatocytes by decreasing lipid secretion, while protecting the body from adverse cardiovascular effects of circulating lipids.^5^ The validation of any interventions predicted from the MOSAICS screen would need to pay particular attention to these outcome-relevant hepatic and extrahepatic consequences, including hepatic fibrosis, hepatocarcinogenesis, circulating lipid profiles and cardiovascular risk.

Nonetheless, understanding cellular adaptations to injury by somatic mutations certainly advances our understanding of NAFLD pathogenesis. In addition, as loss of functional hepatocytes is a common consequence of most liver disorders, it might be interesting to test the MOSAICS platform on other liver diseases, such as genetic disorders, drug toxicity or alcohol-related liver disease, to identify unexpected genes related to hepatocyte fitness in different pathological milieus. While the *Cell* paper by Wang and colleagues represents a fresh and rational approach to drug discovery, its successful translation into novel therapeutics for NAFLD is yet to be demonstrated.
